# Comparison of angiographic change in side-branch ostium after drug-coated balloon vs. drug-eluting stent vs. medication for the treatment of de novo coronary bifurcation lesions

**DOI:** 10.1186/s40001-024-01877-6

**Published:** 2024-05-12

**Authors:** Ae-Young Her, Bitna Kim, Sunwon Kim, Yong Hoon Kim, Bruno Scheller, Eun-Seok Shin

**Affiliations:** 1 Division of Cardiology, Department of Internal Medicine, Kangwon National University College of Medicine, Kangwon National University School of Medicine, Chuncheon, South Korea; 2 Department of Cardiology, Ulsan University Hospital, University of Ulsan College of Medicine, 877 Bangeojinsunhwan-doro, Dong-Gu, Ulsan, 44033 South Korea; 3 Department of Cardiology, Korea University Ansan Hospital, Ansan-Si, South Korea; 4 Universität Des Saarlandes, Campus Homburg, 66421 Homburg, Germany

**Keywords:** Side-branch, Drug-coated balloon, Drug-eluting stent, De novo, Bifurcation lesions, Coronary artery disease

## Abstract

**Objectives:**

Data on side-branch (SB) ostial effect after drug-coated balloon (DCB) treatment in the context of de novo coronary bifurcation lesions are limited. We aimed to investigate the angiographic outcomes of SB ostium after DCB treatment compared with drug-eluting stents (DESs) implantation in the main vessel (MV) or optimal medical therapy (OMT) for the treatment of de novo coronary bifurcation lesions.

**Methods:**

Serial angiographic changes in the SB ostium were compared between DCB, DES, and medication alone for MV treatment. Δ value was calculated by subtracting the follow-up value from the pre-procedure value.

**Results:**

A total of 132 bifurcation lesions were included for analysis (44 lesions in DCB group; 38 lesions in DES group; 50 lesions in OMT group). The minimal lumen diameter (MLD) of SB ostium showed an increase at follow-up in the DCB group, whereas a decrease was observed in both the DES and OMT groups (ΔMLD: −0.16 ± 0.45 mm for DCB group vs. 0.50 ± 0.52 mm for DES group vs. 0.08 ± 0.38 mm for OMT group, *p* < 0.001). The diameter stenosis (DS) of SB ostium showed a marked decrease at follow-up in the DCB group, in contrast to an increase observed in both the DES and OMT groups (ΔDS: 8.01 ± 18.96% for DCB group vs. −18.68 ± 18.60% for DES group vs. −2.05 ± 14.58% for OMT group, *p* < 0.001).

**Conclusions:**

In de novo coronary bifurcation lesions, DCB treatment on the MV demonstrated favorable angiographic outcomes in the SB ostium at 6–9 month follow-up compared to DES implantation or OMT.

**Supplementary Information:**

The online version contains supplementary material available at 10.1186/s40001-024-01877-6.

## Introduction

In the current era of percutaneous coronary intervention (PCI), bifurcation lesion remains a challenging lesion subset which is encountered in up to 20% of all PCI and an independent risk factor for worse clinical outcomes than non-bifurcation lesions [1]. Although previous studies have showed the procedural success and improving efficacy of emerging techniques or devices, most of the evidence is limited to drug-eluting stent (DES) and one of the significant complications for bifurcation PCI is side-branch (SB) occlusion and the most site of in-stent restenosis (ISR) of bifurcation is SB ostium after stenting [2, 3]. Stenting of bifurcation lesions presents certain drawbacks, including the potential for distal vessel overstretching and vessel straightening, both of which can result in a carina or plaque shift into the SB [4]. Despite the appearance of improved flow conditions in the straightened main vessel (MV), the final outcome is actually a compromised situation influenced by the neighboring SB [5].

Because drug-coated balloon (DCB) treatment leaves nothing of lesions behind, it reduces the risk of stent-related adverse biological responses that contribute to restenosis and thrombosis and facilitates favorable natural healing of the vessel [6–9]. Besides delivering an anti-proliferative drug, DCB also contributes to mechanical expansion, resulting in positive vessel remodeling characterized by late lumen enlargement, plaque reduction, and plaque stabilization [10–12]. A previous study demonstrated that DCB treatment of de novo lesions in the MV did not compromise the SB ostium [4]. Instead, it resulted in an increase in the lumen area of the SB ostium after 9-month follow-up. The SB ostial lumen area increased 52.1% (IQR of −0.7% to 77.3%) between post-procedure and 9-month follow-up and 76.1% (IQR 18.2 – 86.6%) between pre-procedure and 9-month follow-up [4]. Nevertheless, there have been no comparative studies between DCB treatment for SB ostium in de novo bifurcation lesions and other treatment modalities like DES implantation or optimal medical therapy (OMT) only without interventional treatment.

Therefore, the aim of this study was to evaluate the effects on SB ostium following DCB treatment compared with DES implantation in the MV or OMT only for de novo coronary bifurcation lesions.

## Methods

### Patient population

Among the patients treated with DCB for de novo coronary artery disease (CAD) included in the DCB registry (Impact of Drug-coated Balloon Treatment in de Novo Coronary Lesion; NCT04619277), a total of 132 patients with de novo bifurcation lesions were retrospectively enrolled from two teaching hospitals in South Korea (Ulsan University Hospital, Ulsan Medical Center). These patients underwent either DCB treatment, DES implantation in the MV, or OMT without interventional treatment. Specifically, when the physician concluded that PCI was not necessary for the bifurcation lesion, OMT was exclusively carried out. The inclusion criteria were bifurcation lesions with a main vessel (MV) diameter of ≥ 3.0 mm and side branch (SB) diameter of ≥ 2.0 mm by visual estimation; however, cases with greater than 50% significant stenosis in the SB ostium were excluded. Exclusion criteria included lesions that require an upfront 2-stent approach or provisional stenting, heavily calcified or thrombotic lesions, chronic total occlusion lesion, left ventricular ejection fraction < 30%, cardiogenic shock, life expectancy < 1 year, and known chronic kidney disease (creatinine > 2 mg/dL). The study protocol received approval from the institutional review board of each participating center, and all patients provided written informed consent at the time of enrollment.

### Procedure

For patients with bifurcation lesions, balloon angioplasty was performed to assess the feasibility of DCB treatment in the MV for PCI. The DCB treatment followed the recommendations of international and Asia–Pacific consensus guidelines for DCB treatment [13–15]. It was mandatory to perform pre-dilatation using a plain balloon with a recommended balloon-to-vessel ratio of 0.8–1.0. After successful pre-dilatation, stenting was delayed for all types of dissections (A to E) if thrombolysis in myocardial infarction (TIMI) grade 3 flow was attained. In cases of flow-limiting dissection after pre-dilatation (TIMI flow grade < 3) or > 30% visual residual stenosis, PCI with stent implantation was recommended. In cases where flow was reduced due to compromise of the SB after the procedure, provisional stenting at SB was performed and was excluded from this study. Therefore, in this study, all patients undergoing DES implantation were managed using the simple crossover 1-stent approach in the MV. All DCBs used were coated with 3.0 μg/mm^2^ paclitaxel combined with iopromide (SeQuent Please^©^ by B. Braun, Germany) as a drug carrier.

### Quantitative coronary angiography (QCA) data

**Fig. 1:**
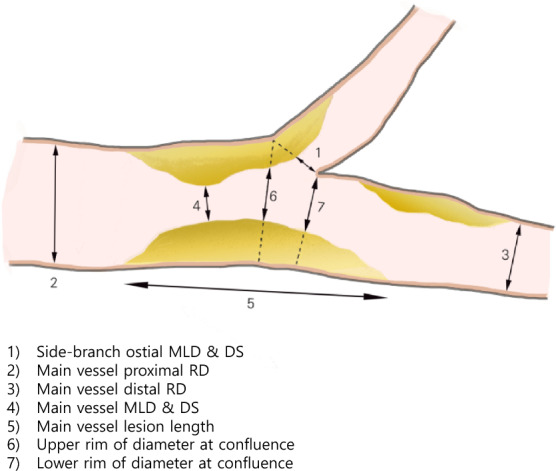
Schematic diagram of quantitative coronary angiographic analysis for bifurcation lesions. *MLD* minimal lumen diameter, *DS* diameter stenosis, *RD* reference diameter

Coronary angiography was performed before and after PCI, during routine 6–9 month angiographic follow-up, and in cases where PCI was deemed necessary due to the presence of new lesions. Quantitative analysis of angiographic data was analyzed offline by a single independent expert in blinded core lab (Cardiovascular Research Foundation in Dong-A University Hospital) using the validated software (Medis Suite XA, Medis, Leiden, The Netherlands). The MV and the SB were assessed separately. To evaluate changes in the SB ostium and the MV, bifurcation lesions were divided into five segments for quantitative coronary angiographic analysis: the SB ostium, proximal MV, distal MV, upper rim of confluence in MV, and lower rim of confluence in MV, as illustrated in Fig. 1. In addition, Δ value was calculated by subtracting the follow-up value from the pre-procedural value.

· Upper rim of confluence in MV: first frame proximally to the take-off of the SB ostium.
· Lower rim of confluence in MV: first frame distally to the take-off of the SB ostium.
· Δminimal lumen diameter (MLD) = (MLD at pre-procedure) – (MLD at follow-up).
· Δdiameter stenosis (DS) = (DS at pre-procedure) – (DS at follow-up).
· ΔUpper rim diameter at confluence = (Upper rim diameter of confluence at pre-procedure) – (Upper rim diameter of confluence at follow-up)
· ΔLower rim diameter of confluence = (Lower rim diameter of confluence at pre-procedure) – (Lower rim diameter of confluence at follow-up)

### Clinical follow-up

All 132 patients underwent a clinical follow-up following the index procedure via telephone interviews and outpatient clinic visits. The major adverse events at 1 year were cardiac death, myocardial infarction (MI), stroke, probable or definite device or stent thrombosis, target vessel revascularization (TVR), and major bleeding. Cardiac death was defined as any death that was not clearly of extracardiac origin, including MI, according to previously published guidelines [16]. Additionally, probable or definite device or stent thrombosis was defined according to the definition by the Academic Research Consortium [17], and major bleeding was defined as Bleeding Academic Research Consortium type 3 to 5 bleeding [18].

### Statistical analysis

For continuous variables, intergroup differences were evaluated using the unpaired *t* test or Mann–Whitney rank test. Analysis of variance (ANOVA) test was used to compare differences of means among three groups. Data are expressed as mean ± standard deviation. For discrete variables, intergroup differences are expressed as count and percentage, and were analyzed with the χ2 or Fisher’s exact test. All p values were two-sided, and values of < 0.05 were considered statistically significant. R version 4.1.2 (R Foundation for Statistical Computing, Vienna, Austria) was used to perform the statistical analysis.

## Results

Among a total of 132 patients with bifurcation lesions, 44 patients (33.3%) composed the DCB group, 38 patients (28.8%) composed the DES group, and 50 patients (37.9%) were received medical therapy alone (OMT group). The baseline clinical and procedural characteristics of the patients are presented in Table 1. Among the three groups, the DCB group exhibited a significantly higher incidence of dyslipidemia compared to the other groups (61.4% for DCB group vs. 31.6% for DES group vs. 40.0% for OMT group; *p* = 0.018). Furthermore, the DCB and DES groups had the highest proportion of patients with unstable angina, whereas the OMT group had mainly stable angina (unstable angina: 56.8% for DCB group vs. 63.2% for DES group vs. 38.0% for OMT group; stable angina: 34.1% for DCB group vs. 21.0% for DES group vs. 44.0% for OMT group, *p* = 0.021).

**Table 1:** Baseline clinical and procedural characteristics of the patients

Variables	DCB	DES	Medication	*P* value	*P* value for DCB vs. DES
	(*n* = 44)	(*n* = 38)	(*n* = 50)		
Age, years	61.6 ± 9.0	63.0 ± 9.7	61.0 ± 8.8	0.583	0.504
Male, *n* (%)	32 (72.7)	27 (71.1)	30 (60.0)	0.360	> 0.999
LV ejection fraction, %	62.6 ± 5.2	61.0 ± 6.4	60.6 ± 8.8	0.390	0.228
Cardiovascular risk factors, *n* (%)					
Hypertension	30 (68.2)	22 (57.9)	30 (60.0)	0.585	0.463
Diabetes mellitus	16 (36.4)	14 (36.8)	18 (36.0)	0.997	> 0.999
Dyslipidemia	27 (61.4)	12 (31.6)	20 (40.0)	0.018	0.013
Current smoker	9 (20.5)	10 (27.0)	19 (38.0)	0.166	0.666
Family history of CAD	8 (18.2)	4 (10.5)	16 (32.0)	0.082	0.383
Clinical diagnosis, *n* (%)				0.021	0.029
Stable angina	15 (34.1)	8 (21.0)	22 (44.0)		
Unstable angina	25 (56.8)	24 (63.2)	19 (38.0)		
NSTEMI	4 (9.1)	3 (7.9)	5 (10.0)		
STEMI	0	3 (7.9)	4 (8.0)		
Angiographic findings					
Location of bifurcation, *n* (%)				0.238	0.490
LM	8 (18.2)	10 (26.3)	7 (14.0)		
LAD	21 (47.7)	20 (52.6)	22 (44.0)		
LCX	9 (20.5)	6 (15.8)	12 (24.0)		
RCA	6 (13.6)	2 (5.3)	9 (18.0)		
Type of bifurcation lesion by Medina classification, *n* (%)				0.242	0.927
0,0,1	4 (9.1)	3 (7.9)	12 (24.0)		
0,1,0	12 (27.3)	12 (31.6)	20 (40.0)		
0,1,1	4 (9.1)	6 (15.8)	5 (10.0)		
1,0,0	7 (15.9)	5 (13.2)	2 (4.0)		
1,0,1	2 (4.5)	1 (2.6)	2 (4.0)		
1,1,0	6 (13.6)	6 (15.8)	6 (12.0)		
1,1,1	9 (20.5)	5 (13.2)	3 (6.0)		
True bifurcation lesion, n (%)	15 (34.1)	12 (31.6)	10 (20.0)	0.268	0.995

Values are presented as the mean ± SD or n (%)

*DCB* drug-coated balloon, *DES* drug-eluting stent, *LV* left ventricular, *CAD* coronary artery disease, *NSTEMI* non-ST segment elevation myocardial infarction, *STEMI* ST-segment elevation myocardial infarction, *LM* left main, *LAD* left anterior descending artery, *LCX* left circumflex artery, *RCA* right coronary artery

**Fig. 2:**
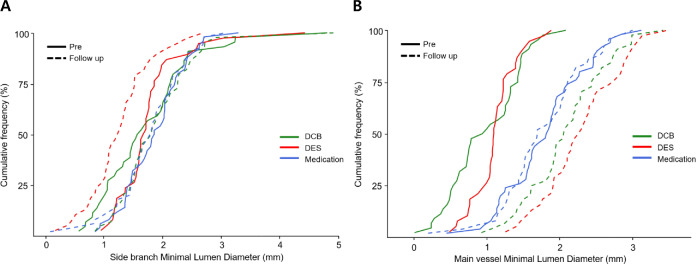
Cumulative frequency distribution of angiographic change of (**A**) MLD in the SB ostium and (**B**) MLD in the MV between pre-procedure and 6–9 month follow-up according to treatment strategy. *DCB* drug-coated balloon, *DES* drug-eluting stent

**Fig. 3:**
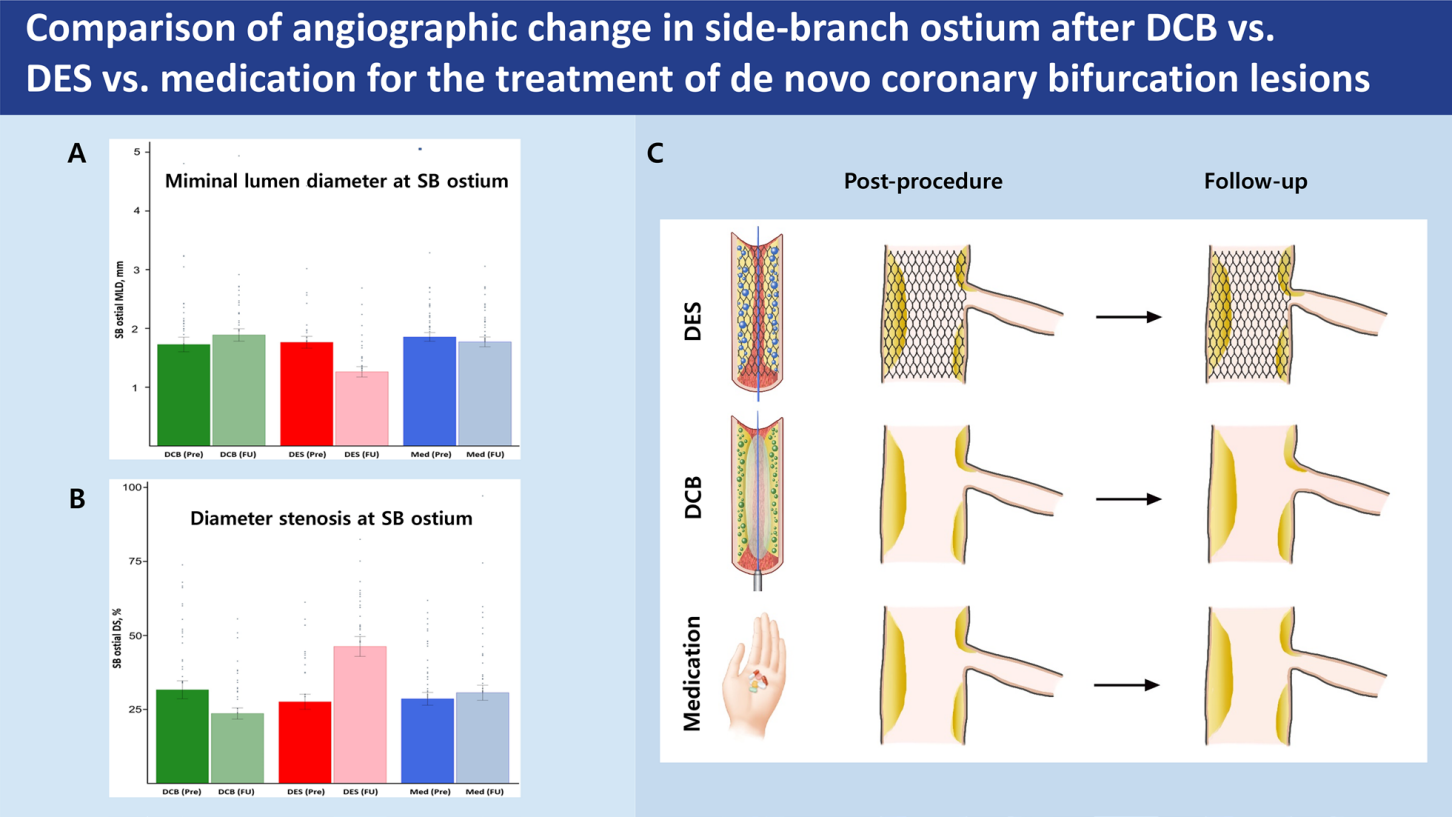
Central illustration. The changes of (**A**) MLD and (**B**) DS in the SB ostium between pre-procedure and 6–9 month follow-up according to treatment strategy. (**C**) DCB treatment on the MV only in patients with de novo coronary bifurcation lesions demonstrated superior angiographic outcomes in the SB ostium compared with DES implantation or OMT alone after 6–9 month follow-up

The QCA data are showed in Table 2 and Supplementary Table 1. The MLD of SB ostium showed an increase at 6–9 month follow-up in the DCB group, whereas a decrease was observed in both the DES and OMT groups (ΔMLD: −0.16 ± 0.45 mm for DCB group vs. 0.50 ± 0.52 mm for DES group vs. 0.08 ± 0.38 mm for OMT group, *p* < 0.001) (Figs. 2A and 3A and Central Illustration Fig. 3C). The DS of SB ostium showed a marked decrease at 6–9 month follow-up in the DCB group, in contrast to an increase observed in both the DES and OMT groups (ΔDS: 8.0 ± 19.0% for DCB group vs. −18.7 ± 18.6% for DES group vs. −2.1 ± 14.6% for OMT group, *p* < 0.001) (Fig, 3B, C). The MLD in the MV showed an increase at 6–9 month follow-up in both the DCB and DES groups, while a decrease was observed in the OMT group (ΔMLD: −1.11 ± 0.58 mm for DCB group vs. −1.18 ± 0.50 mm for DES group vs. 0.03 ± 0.27 mm for OMT group, *p* < 0.001) (Fig. 2B and Supplementary Fig. 1A). The DS in the MV showed a marked decrease at 6–9 month follow-up in both the DCB and DES groups, contrasting with a smaller decrease observed in the OMT group (ΔDS: 38.5 ± 19.0% for DCB group vs. 35.7 ± 13.5% for DES group vs. 0.7 ± 8.6% for OMT group, *p* < 0.001) (Supplementary Fig. 1B). The upper and lower rim diameter of confluence in the MV also showed increases at 6–9 month follow-up in both the DCB and DES groups, while decreases were observed in the OMT group (Δupper rim: −0.76 ± 0.79 mm for DCB group vs. −0.68 ± 0.70 mm for DES group vs. 0.11 ± 0.30 mm for OMT group, *p* < 0.001; Δlower rim: −0.75 ± 0.66 mm for DCB group vs. −0.83 ± 0.69 mm for DES group vs. 0.01 ± 0.29 mm for OMT group, *p* < 0.001). The results obtained from the analysis of true bifurcation lesions in Supplementary Table 1 were consistent with those of all bifurcation lesions in Table 2. In true bifurcation lesions, compared to all bifurcation lesions, the pre-procedural MLD of the SB ostium was smaller and the DS was more severe. However, after DCB treatment, the absolute values of ΔMLD and ΔDS were actually greater after follow-up.

**Table 2:** Quantitative coronary angiography measurements

Variables	DCB	DES	Medication	*P* value	*P* value for DCB vs. DES
	(*n* = 44)	(*n* = 38)	(*n* = 50)		
Side-branch ostium					
* Pre-procedure*					
MLD, mm	1.73 ± 0.83	1.77 ± 0.62	1.86 ± 0.53	0.631	0.820
DS, %	31.7 ± 20.1	27.7 ± 15.7	28.7 ± 15.3	0.531	0.319
*Post-procedure*					
MLD, mm	1.88 ± 0.68	1.41 ± 0.57	–	–	0.001
DS, %	30.5 ± 17.2	42.4 ± 20.6	–	–	0.006
*6–9 month follow-up*					
MLD, mm	1.89 ± 0.70	1.26 ± 0.55	1.77 ± 0.59	< 0.001	< 0.001
DS, %	23.7 ± 12.6	46.4 ± 20.6	30.7 ± 18.0	< 0.001	< 0.001
ΔMLD, mm	−0.16 ± 0.45	0.50 ± 0.52	0.08 ± 0.38	< 0.001	< 0.001
ΔDS, %	8.0 ± 19.0	−18.7 ± 18.6	−2.1 ± 14.6	< 0.001	< 0.001
Main vessel					
* Pre-procedure*					
RD, mm	2.84 ± 0.52	2.77 ± 0.45	2.92 ± 0.63	0.414	0.483
Lesion length, mm	20.86 ± 6.55	28.20 ± 11.22	14.01 ± 6.11	< 0.001	0.001
MLD, mm	0.97 ± 0.50	1.11 ± 0.31	1.80 ± 0.57	< 0.001	0.126
DS, %	66.8 ± 14.4	59.9 ± 8.4	39.0 ± 12.2	< 0.001	0.009
Upper rim diameter at confluence, mm	2.17 ± 0.97	2.42 ± 0.70	2.72 ± 0.75	0.006	0.182
Lower rim diameter at confluence, mm	1.86 ± 0.63	2.00 ± 0.68	2.10 ± 0.60	0.176	0.345
*Post-procedure*					
MLD, mm	2.25 ± 0.36	2.67 ± 0.40	–	–	< 0.001
DS, %	24.6 ± 8.1	15.6 ± 7.1	–	–	< 0.001
Upper rim diameter at confluence, mm	3.01 ± 0.57	3.25 ± 0.49	–	–	0.042
Lower rim diameter at confluence, mm	2.73 ± 0.46	3.07 ± 0.46	–	–	0.001
*6–9 month follow-up*					
MLD, mm	2.09 ± 0.56	2.29 ± 0.54	1.78 ± 0.58	< 0.001	0.092
DS, %	28.3 ± 13.4	24.3 ± 12.5	38.3 ± 14.4	< 0.001	0.164
Upper rim diameter at confluence, mm	2.94 ± 0.71	3.10 ± 0.49	2.61 ± 0.74	0.002	0.225
Lower rim diameter at confluence, mm	2.61 ± 0.63	2.83 ± 0/48	2.09 ± 0.60	< 0.001	0.083
ΔMLD, mm	−1.11 ± 0.58	−1.18 ± 0.50	0.03 ± 0.27	< 0.001	0.579
ΔDS, %	38.5 ± 19.0	35.7 ± 13.5	0.7 ± 8.6	< 0.001	0.430
ΔUpper rim diameter at confluence, mm	−0.76 ± 0.79	−0.68 ± 0.70	0.11 ± 0.30	< 0.001	0.607
ΔLower rim diameter at confluence, mm	−0.75 ± 0.66	−0.83 ± 0.69	0.01 ± 0.29	< 0.001	0.581

Values are presented as the mean ± SD

*DCB* drug-coated balloon, *DES* drug-eluting stent, *MLD* minimal lumen diameter, *RD* reference diameter, *DS* diameter stenosis

No additional bailout stenting, SB occlusion, or slow flow (TIMI < 3) occurred after DCB treatment. During the 1-year follow-up period, there were no significant major adverse events except for 2 cases of TVR in each of the DES and OMT groups.

## Discussion

This study provided that DCB treatment of MV alone on de novo coronary bifurcation lesions resulted in an increase in the SB ostial lumen diameter and a decrease in the SB ostial stenosis at 6–9 month follow-up, as compared to DES implantation or OMT therapy.

DES implantation in bifurcation lesions, as with any current stenting strategy, has an impact on the regional arterial geometry, thereby causing modifications to local flow conditions and damages to the SB ostium [19, 20]. Hahn et al. demonstrated that after DES implantation for bifurcation lesions, 8.4% of patients experienced SB occlusion, and those with SB occlusion had a higher incidence of cardiac death or MI compared to those without SB occlusion (aHR: 2.34; 95% CI 1.15 to 4.77; *p* = 0.02) [21]. These stent-induced alterations of blood flow result in complex spatiotemporal modifications in wall shear stress, which subsequently enhance the thrombogenicity around the stent struts and alterations in the endothelial phenotype that facilitate the migration of inflammatory cells [22]. If plaque burden within the bifurcation lesions is adequately reduced and the arterial geometry and blood flow are maintained without the inclusion of foreign materials like stent struts, it would represent an ideal bifurcation PCI.

DCB may offer several advantages over DES by ensuring immediate and homogeneous drug uptake by the vessel wall, without the potential for inflammatory reactions associated with stent struts or polymers, and maintaining the original anatomy of the bifurcation [23]. To date, previous bifurcation studies for DCB treatment have primarily examined the results of using DCB sequentially on the MV and SB while stenting the MV [24–27]. However, these studies have been limited in accurately assessing the direct impact on the SB ostium following DCB treatment in bifurcation lesions, primarily due to the presence of a stent in the MV. Almost exclusively, Her et al. demonstrated the benefits of avoiding carina shift and maintaining the natural distribution of blood flow through an increase in the SB ostium when applying a DCB-only approach to the MV [4]. Nevertheless, it should be noted that the study was a single-arm trial and did not directly compare the approach with stenting.

This study is the first to demonstrate a difference in the serial angiographic change of SB ostium according to the treatment strategy on MV including DCB-only approach, DES implantation, or OMT alone. In this study, the comparison of the angiographic changes in the SB ostium from baseline to follow-up revealed that the SB ostium in the DCB group markedly increased, whereas in the DES and OMT groups tended to decrease. Therefore, these findings of this study provide direct evidence that the effect of DCB application on the MV in bifurcation lesions, which extends to the SB ostium, can be beneficial in reducing complications associated with the SB.

The specific finding by which DCB treatment leads to the enlargement of the SB ostium adjacent to the MV is not well-established, it may be explained that DCB does not have the restrictions of stents that inhibit vessel enlargement, potentially facilitating an increase in vessel diameter [28–30]. When pre-dilatation creates a deep dissection that extends into the tunica media, the tunica media becomes exposed to the vessel lumen, which can result in vascular enlargement [31]. Additionally, it may be a result of positive vessel remodeling caused by the localized drug delivery effects from DCB treatment [31, 32]. Despite the absence of a metal foreign body such as DES in the MV, the OMT group did not show any changes in the SB ostium. Conversely, the DCB group exhibited an enlarging effect on the SB ostium, suggesting a possible explanation.

Our study has several limitations that should be taken into account. First, it was an observational study conducted with a limited number of patients and lesions. Nonetheless, we made efforts to conduct a meticulous analysis by a single independent expert from a blinded core lab to overcome this limitation. Second, between-group bias appears to exist as the treatment strategy was determined by physicians. DES was implanted only in cases where DCB was deemed unacceptable following pre-dilatation. Additionally, due to the limited use of intravascular imaging during follow-up, precise assessments of vessel area or plaque area were not available. However, to report the changes in the SB ostium objectively based on real clinical practice, we utilized QCA according to the treatment strategy. Further validation studies are necessary to confirm the effect of plaque regression and vessel enlargement associated with DCB treatment on bifurcation lesion. It should also be revealed whether such results lead to improved outcomes.

## Conclusions

DCB treatment on the MV in patients with de novo coronary bifurcation lesions showed better angiographic outcomes in the SB ostium compared to DES implantation or OMT alone after the 6–9 month follow-up.

### Supplementary Information

Supplementary Materials 1: Figure 1. Changes of (A) MLD and (B) DS in the MV between pre-procedure and 6–9 month follow-up according to treatment strategy. MLD: minimal lumen diameter; DS: diameter stenosis; SB: side-branch; MV: main vessel.

Supplementary Materials 2: Table S1 Quantitative coronary angiography measurements for true bifurcation lesions (Medina 1,1,1 or 0,1,1 or 1,0,1 lesions).

## Data Availability

The datasets used and/or analyzed during the current study are available from the corresponding author on reasonable request.

## Abbreviations

CAD: Coronary artery disease
DCB: Drug-coated balloon
DES: Drug-eluting stent
OMT: Optimal medical therapy
DS: Diameter stenosis
MLD: Minimal lumen diameter
MV: Main vessel
PCI: Percutaneous coronary intervention
SB: Side-branch

## References

[1] Sawaya FJ, Lefevre T, Chevalier B, Garot P, Hovasse T, Morice MC, Rab T, Louvard Y (2016). Contemporary approach to coronary bifurcation lesion treatment. JACC Cardiovasc Interv.

[2] Burzotta F, Lassen JF, Lefevre T, Banning AP, Chatzizisis YS, Johnson TW, Ferenc M, Rathore S, Albiero R, Pan M, Darremont O, Hildick-Smith D, Chieffo A, Zimarino M, Louvard Y, Stankovic G (2021). Percutaneous coronary intervention for bifurcation coronary lesions: the 15^th^ consensus document from the European bifurcation club. EuroIntervention.

[3] Gil RJ, Vassilev D, Formuszewicz R, Rusicka-Piekarz T, Doganov A (2009). The carina angle-new geometrical parameter associated with periprocedural side branch compromise and the long-term results in coronary bifurcation lesions with main vessel stenting only. J Interv Cardiol.

[4] Her AY, Ann SH, Singh GB, Kim YH, Okamura T, Garg S, Koo BK, Shin ES (2016). Serial morphological changes of side-branch ostium after paclitaxel-coated balloon treatment of de novo coronary lesions of main vessels. Yonsei Med J.

[5] Dou K, Zhang D, Xu B, Yang Y, Yin D, Qiao S, Wu Y, Yan H, You S, Wang Y, Wu Z, Gao R, Kirtane AJ (2015). An angiographic tool for risk prediction of side branch occlusion in coronary bifurcation intervention: the RESOLVE score system (risk prediction of side branch occlusion in coronary bifurcation intervention). JACC Cardiovasc Interv.

[6] Ann SH, Balbir Singh G, Lim KH, Koo BK, Shin ES (2016). Anatomical and physiological changes after paclitaxel-coated balloon for atherosclerotic de novo coronary lesions: serial IVUS-VH and FFR study. PLoS ONE.

[7] Shin ES, Jun EJ, Kim S, Kim B, Kim TH, Sohn CB, Her AY, Park Y, Cho JR, Jeong YH, Choi BJ (2023). Clinical impact of drug-coated balloon-based percutaneous coronary intervention in patients with multivessel coronary artery disease. JACC Cardiovasc Interv.

[8] Her AY, Kim B, Ahn SH, Park Y, Cho JR, Jeong YH, Shin ES (2023). Long-term clinical outcomes of drug-coated balloon treatment for de novo coronary lesions. Yonsei Med J.

[9] Her AY, Yuan SL, Jun EJ, Bhak Y, Kim MH, Garg S, Kim YH, Kun L, Hui L, Zhi W, Hao J, Zhentao S, Qiang T, Shin ES (2021). Drug-coated balloon treatment for nonsmall de-novo coronary artery disease: angiographic and clinical outcomes. Coron Artery Dis.

[10] Her AY, Shin ES, Chung JH, Kim YH, Garg S, Lee JM, Doh JH, Nam CW, Koo BK (2019). Plaque modification and stabilization after paclitaxel-coated balloon treatment for de novo coronary lesions. Heart Vessels.

[11] Her AY, Ann SH, Singh GB, Kim YH, Yoo SY, Garg S, Koo BK, Shin ES (2016). Comparison of paclitaxel-coated balloon treatment and plain old balloon angioplasty for de novo coronary lesions. Yonsei Med J.

[12] Scheller B, Fischer D, Clever YP, Kleber FX, Speck U, Bohm M, Cremers B (2013). Treatment of a coronary bifurcation lesion with drug-coated balloons: lumen enlargement and plaque modification after 6 months. Clin Res Cardiol.

[13] Jeger RV, Eccleshall S, Wan Ahmad WA, Ge J, Poerner TC, Shin ES, Alfonso F, Latib A, Ong PJ, Rissanen TT, Saucedo J, Scheller B, Kleber FX (2020). Drug-coated balloons for coronary artery disease: third report of the international DCB consensus group. JACC Cardiovasc Interv.

[14] Her AY, Shin ES, Bang LH, Nuruddin AA, Tang Q, Hsieh IC, Hsu JC, Kiam OT, Qiu C, Qian J, Ahmad WAW, Ali RM (2021). Drug-coated balloon treatment in coronary artery disease: recommendations from an Asia-pacific consensus group. Cardiol J.

[15] Shin ES, Bang LH, Jun EJ, Her AY, Chung JH, Garg S, Lee JM, Doh JH, Nam CW, Koo BK, Tang Q (2021). Provisional drug-coated balloon treatment guided by physiology on de novo coronary lesion. Cardiol J.

[16] Yahagi K, Kolodgie FD, Otsuka F, Finn AV, Davis HR, Joner M, Virmani R (2016). Pathophysiology of native coronary, vein graft, and in-stent atherosclerosis. Nat Rev Cardiol.

[17] Cutlip DE, Windecker S, Mehran R, Boam A, Cohen DJ, van Es GA, Steg PG, Morel MA, Mauri L, Vranckx P, McFadden E, Lansky A, Hamon M, Krucoff MW, Serruys PW (2007). Clinical end points in coronary stent trials: a case for standardized definitions. Circulation.

[18] Mehran R, Rao SV, Bhatt DL, Gibson CM, Caixeta A, Eikelboom J, Kaul S, Wiviott SD, Menon V, Nikolsky E, Serebruany V, Valgimigli M, Vranckx P, Taggart D, Sabik JF, Cutlip DE, Krucoff MW, Ohman EM, Steg PG, White H (2011). Standardized bleeding definitions for cardiovascular clinical trials: a consensus report from the bleeding academic research consortium. Circulation.

[19] Sheiban I, Figini F, Gasparetto V, D'Ascenzo F, Moretti C, Leonardo F (2021). Side branch is the main determinant factor of bifurcation lesion complexity: critical review with a proposal based on single-centre experience. Heart Int.

[20] Genuardi L, Chatzizisis YS, Chiastra C, Sgueglia G, Samady H, Kassab GS, Migliavacca F, Trani C, Burzotta F (2021). Local fluid dynamics in patients with bifurcated coronary lesions undergoing percutaneous coronary interventions. Cardiol J.

[21] Hahn JY, Chun WJ, Kim JH, Song YB, Oh JH, Koo BK, Rha SW, Yu CW, Park JS, Jeong JO, Choi SH, Choi JH, Jeong MH, Yoon JH, Jang Y, Tahk SJ, Kim HS, Gwon HC (2013). Predictors and outcomes of side branch occlusion after main vessel stenting in coronary bifurcation lesions: results from the COBIS II registry (coronary bIfurcation stenting). J Am Coll Cardiol.

[22] Otsuka F, Finn AV, Yazdani SK, Nakano M, Kolodgie FD, Virmani R (2012). The importance of the endothelium in atherothrombosis and coronary stenting. Nat Rev Cardiol.

[23] Herdeg C, Oberhoff M, Baumbach A, Blattner A, Axel DI, Schroder S, Heinle H, Karsch KR (2000). Local paclitaxel delivery for the prevention of restenosis: biological effects and efficacy in vivo. J Am Coll Cardiol.

[24] Stella PR, Belkacemi A, Dubois C, Nathoe H, Dens J, Naber C, Adriaenssens T, van Belle E, Doevendans P, Agostoni P (2012). A multicenter randomized comparison of drug-eluting balloon plus bare-metal stent versus bare-metal stent versus drug-eluting stent in bifurcation lesions treated with a single-stenting technique: six-month angiographic and 12-month clinical results of the drug-eluting balloon in bifurcations trial. Catheter Cardiovasc Interv.

[25] Berland J, Lefevre T, Brenot P, Fajadet J, Motreff P, Guerin P, Dupouy P, Schandrin C (2015). DANUBIO—a new drug-eluting balloon for the treatment of side branches in bifurcation lesions: six-month angiographic follow-up results of the DEBSIDE trial. EuroIntervention.

[26] Jim MH, Lee MK, Fung RC, Chan AK, Chan KT, Yiu KH (2015). Six month angiographic result of supplementary paclitaxel-eluting balloon deployment to treat side branch ostium narrowing (SARPEDON). Int J Cardiol.

[27] Worthley S, Hendriks R, Worthley M, Whelan A, Walters DL, Whitbourn R, Meredith I (2015). Paclitaxel-eluting balloon and everolimus-eluting stent for provisional stenting of coronary bifurcations: 12-month results of the multicenter BIOLUX-I study. Cardiovasc Revasc Med.

[28] Jun EJ, Yuan SL, Garg S, Shin ES (2020). Serial optical coherence tomography findings after drug-coated balloon treatment in de novo coronary bifurcation lesion. Cardiol J.

[29] Kim TH, Chung JH, Shin ES (2020). A case of drug-coated balloon treatment for three-vessel stenosis with left main bifurcation lesion. Cardiol J.

[30] Yuan SL, Jun EJ, Kim MH, Shin ES (2021). Late lumen enlargement and plaque regression after drug-coated balloon treatment for an isolated ostial lesion of a diagonal branch. Cardiol J.

[31] Sogabe K, Koide M, Fukui K, Kato Y, Kitajima H, Akabame S, Zen K, Nakamura T, Matoba S (2021). Optical coherence tomography analysis of late lumen enlargement after paclitaxel-coated balloon angioplasty for de-novo coronary artery disease. Catheter Cardiovasc Interv.

[32] Kleber FX, Schulz A, Waliszewski M, Hauschild T, Bohm M, Dietz U, Cremers B, Scheller B, Clever YP (2015). Local paclitaxel induces late lumen enlargement in coronary arteries after balloon angioplasty. Clin Res Cardiol.

