# Supplementary figures and images for: Comparison of angiographic change in side-branch ostium after drug-coated balloon vs. drug-eluting stent vs. medication for the treatment of de novo coronary bifurcation lesions
Source: Eur J Med Res. 2024 May 12;29:280. doi: 10.1186/s40001-024-01877-6 (PMC11089776; doi:10.1186/s40001-024-01877-6)

## Slide 1
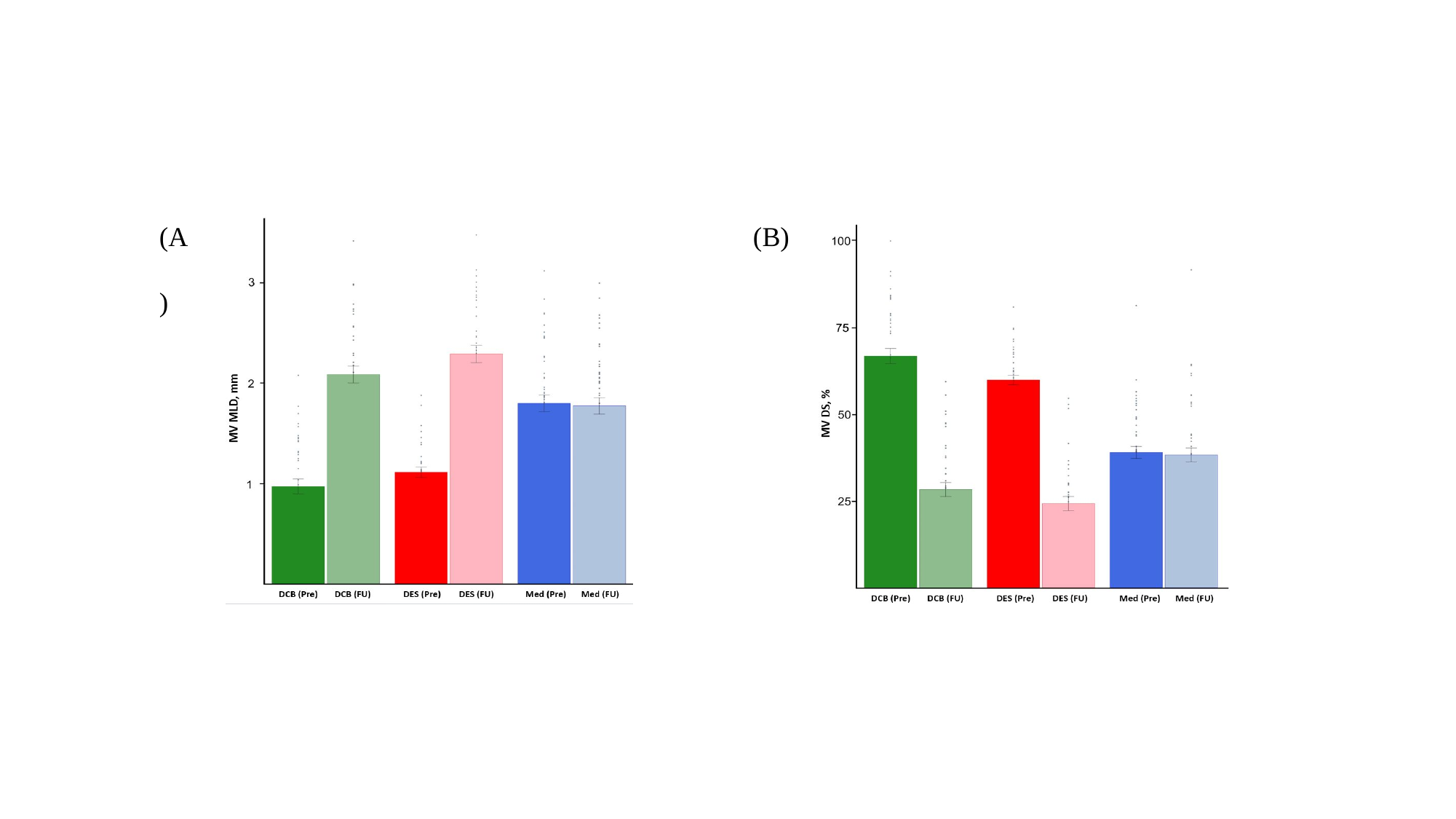

(A)
(B)

Supplement: Supplementary file 1 — Supplementary Materials 1: Figure 1. Changes of (A) MLD and (B) DS in the MV between pre-procedure and 6–9 month follow-up according to treatment strategy. MLD: minimal lumen diameter; DS: diameter stenosis; SB: side-branch; MV: main vessel. [file 40001_2024_1877_MOESM1_ESM.pptx]
