# Supplementary material for: Comparison of angiographic change in side-branch ostium after drug-coated balloon vs. drug-eluting stent vs. medication for the treatment of de novo coronary bifurcation lesions
Source: Eur J Med Res. 2024 May 12;29:280. doi: 10.1186/s40001-024-01877-6 (PMC11089776; doi:10.1186/s40001-024-01877-6)
Supplement: Supplementary file 2 — Supplementary Materials 2: Table S1 Quantitative coronary angiography measurements for true bifurcation lesions (Medina 1,1,1 or 0,1,1 or 1,0,1 lesions). [file 40001_2024_1877_MOESM2_ESM.doc]

**Supplementary Table S1** Quantitative coronary angiography measurements for true bifurcation lesions (Medina 1,1,1 or 0,1,1 or 1,0,1 lesions)

| Variables | DCB | DES | Medication | | P value | P value for  DCB vs DES |
| --- | --- | --- | --- | --- | --- | --- |
|  | (n = 15) | (n = 12) | (n = 10) | |
| **Side-branch ostium** |  |  |  | |  |  |
| *Pre-procedure* |  |  |  | |  |  |
| MLD, mm | 1.15 ± 0.41 | 1.48 ± 0.30 | 1.70 ± 0.67 | | 0.019 | 0.029 |
| DS, % | 49.19 ± 13.28 | 39.39 ± 14.78 | 40.57 ± 14.14 | | 0.155 | 0.082 |
| *6-9 months follow-up* |  |  |  | |  |  |
| MLD, mm | 1.52 ± 0.39 | 1.11 ± 0.45 | 1.83 ± 0.61 | | 0.005 | 0.018 |
| DS, % | 28.86 ± 13.32 | 55.62 ± 16.33 | 37.66 ± 11.09 | | <0.001 | <0.001 |
| ΔMLD, mm | -0.37 ± 0.39 | 0.36 ± 0.49 | -0.12 ± 0.25 | | <0.001 | <0.001 |
| ΔDS, % | 20.33 ± 18.52 | -16.22 ± 21.10 | 2.91 ± 8.56 | | <0.001 | <0.001 |
| **Main vessel** |  |  |  | |  |  |
| *Pre-procedure* |  |  |  | |  |  |
| RD, mm | 2.78 ± 0.51 | 3.00 ± 0.44 | | 3.21 ± 0.86 | 0.236 | 0.249 |
| Lesion length, mm | 20.71 ± 7.10 | 27.92 ± 10.03 | | 14.02 ± 4.95 | 0.001 | 0.038 |
| MLD, mm | 0.81 ± 0.41 | 1.22 ± 0.36 | 1.71 ± 0.77 | | 0.001 | 0.012 |
| DS, % | 71.05 ± 13.97 | 59.67 ± 8.70 | 48.34 ± 15.32 | | 0.001 | 0.021 |
| Upper rim diameter at confluence, mm | 1.49 ± 0.67 | 2.24 ± 0.94 | 2.68 ± 1.07 | | 0.006 | 0.022 |
| Lower rim diameter at confluence, mm | 1.44 ± 0.58 | 1.98 ± 0.99 | 2.07 ± 0.72 | | 0.092 | 0.091 |
| *6-9 months follow-up* |  |  |  | |  |  |
| MLD, mm | 2.04 ± 0.60 | 2.51 ± 0.54 | 1.56 ± 0.81 | | 0.006 | 0.046 |
| DS, % | 25.49 ± 14.79 | 21.92 ± 9.58 | 52.98 ± 17.80 | | <0.001 | 0.477 |
| Upper rim diameter at confluence, mm | 2.61 ± 0.67 | 3.28 ± 0.57 | 2.57 ± 1.07 | | 0.053 | 0.011 |
| Lower rim diameter at confluence, mm | 2.44 ± 0.69 | 3.02 ± 0.47 | 2.14 ± 0.77 | | 0.010 | 0.021 |
| ΔMLD, mm | -1.23 ± 0.68 | -1.28 ± 0.55 | 0.15 ± 0.14 | | <0.001 | 0.820 |
| ΔDS, % | 45.56 ± 20.85 | 37.76 ± 11.59 | -4.64 ± 3.98 | | <0.001 | 0.257 |
| ΔUpper rim diameter at confluence, mm | -1.13 ± 0.82 | -1.03 ± 0.84 | 0.11 ± 0.24 | | <0.001 | 0.778 |
| ΔLower rim diameter at confluence, mm | -1.00 ± 0.65 | -1.04 ± 0.89 | -0.06 ± 0.16 | | 0.002 | 0.881 |

Values are presented as the mean ± SD.

DCB, drug-coated balloon; DES, drug eluting stent; MLD, minimal lumen diameter; DS, diameter stenosis; RD, reference diameter.
